# Differential Expression Pattern of Epithelial Mesenchymal Transition Gens: AXL, GAS6, Claudin-1, and Cofilin-1, in Different Stages of Epithelial Ovarian Cancer

**Published:** 2019-09

**Authors:** Elham HASSANI, Mahmood SHEKARI KHANIANI, Mojtaba SAFFARI, Amirnader EMAMI RAZAVI, Reza SHIRKOOHI, Sima MANSOORI DERAKHSHAN

**Affiliations:** 1. Department of Medical Genetics, Faculty of Medicine, Tabriz University of Medical Sciences, Tabriz, Iran; 2. Cancer Biology Research Center, Cancer Institute of Iran, Tehran University of Medical Sciences, Tehran, Iran; 3. Iran National Tumor Bank, Cancer Institute of Iran, Tehran University of Medical Sciences, Tehran, Iran; 4. Immunology Research Center, Tabriz University of Medical Sciences, Tabriz, Iran

**Keywords:** QRT-PCR, AXL, GAS6, Claudin-1, Cofilin-1

## Abstract

**Background:**

Epithelial ovarian cancer (EOC), is the fatal form of gynecological cancer. Almost 70% of ovarian cancer patients are detected at an advanced stage (III–IV) with metastases. Epithelial‐mesenchymal transition (EMT) is a critical process associated with metastasis. This study investigated the expression levels of AXL, GAS6, Claudin-1, and Cofilin-1, as genes involved in EMT in relation to clinicopathologic features in ovarian cancer patients.

**Methods:**

In this descriptive study, 78 ovarian epithelial cancer patients were enrolled. Samples were provided by the Iran National Tumor Bank, founded by the Cancer Institute of Tehran University of Medical Sciences in 2017. The expression levels of AXL, GAS6, Claudin-1, and Cofilin-1 genes were investigated in a fresh, frozen tumor sample and normal adjacent tissue by real-time PCR (RT-PCR).

**Results:**

Findings showed a significant relationship between the overexpression of AXL and TNM staging (*P*=0.03). The expression level of GAS6 decreased in more advanced stages (*P*=0.01). There is a negative relationship between Cofilin-1 expression level and TNM staging (*P*=0.002). Claudin-1 expression level was higher in low stages compared with that in high stages (*P*=0.01). There was no relationship between gene expression levels of target genes with size and grade of the tumor.

**Conclusion:**

Given the importance of these genes in EMT, alteration in their expression pattern can contribute to the progression of the disease and distant metastasis of cancer cells. Additionally, knowing the alteration pattern of these genes expression can help to better understanding and prediction of the prognosis of EOC.

## Introduction

Ovarian cancer is fifth reason in cancer dependent diseases among females (1). Epithelial ovarian cancer (EOC), correlated with 90% of all ovarian cancers, is the most prevalent and fatal form of gynecological cancer in developed countries (2). In Iran, ovarian cancer is the 8th prevalent cancer type (3). The usual therapy for ovarian cancer contains reduction of cells after platinum chemotherapy. Despite an initial response to the treatment, due to the presence of chemotherapy-resistant residual tumor cells, many patients will be finally affected by metastasis and eventually will die (4). Since ovarian cancer develops without any apparent symptoms (5), almost 70% of ovarian cancer patients are detected at an advanced stage (III–IV) with metastases, of which only 45% survive five years after initial diagnosis (6). Probable reasons for this poor prognosis include lack of screening instruments for early-stage diagnosis, the nonspecific symptoms, and drug resistance in advanced disease. Metastasis in cancer is the most challenging problem in clinical issue (7). Metastasis in cancer is the cause of 90% of all cancer-associated die, since the biologic and physical factors that define the final form and heterogenicity of tumors with metastasis are not yet well understood (8). In fact, 90% of all ovarian cancers are EOC that originates from the ovarian surface epithelium (OSE). The transformation of epithelial cells towards motile mesenchymal state is a pivotal process associated with metastasis named epithelial-to-mesenchymal transition (EMT) (9).

There are three different forms of EMT; Type 3 happen in cancer (metastasis) (10). In cancerous epithelial cells, cell junctions and apical-basal polarity are disappeared, cell cytoskeleton is rearranged, signaling programs that establish cell shape and alter gene expression program are changed, during EMT. These alterations result in enhanced cell movement and gain of invasive manifestation by cancer cells (11). Thus, attempts to identify molecular targets regulating ovarian metastasis are critical for early detection and treatment of EOC (12). With respect to the steps of EMT, which are essential for metastasis, the researchers of the present study studied a number of genes reported to have a role in EMT in different cancers, such as Claudin-1, Cofilin-1, AXL, and GAS6.

Tight Junctions have vital roles in controlling paracellular transport and in keeping cell polarity (13). Claudins are a member of cellular binding molecules that are ingredients of tight junctions, which have crucial roles in cell polarity and cell transport (14). The Claudins are a group of over 20 proteins that expression is tissue-specific (15). Different pieces of findings offer that Claudin-1 is a crucial ingredient of TJs and is directly have a role in the fence task of TJs. Claudin-1 is located at 3q28-q29 (16). As is known, destroy of the cell-cell junction is an important phase in EMT; in this regard, claudin-1 has capability to directly induce EMT via its interaction with specified EMT-associated transcription factors and signaling pathways (17).

Numerous pieces of evidence suggest the important function of the cytoskeleton in the EMT (9). Actin cytoskeleton and its interaction proteins are remodeled and are the motive power for cell migrate and invasiveness of malignant cancer cells (18). Cofilin (CFL), is an F-actin-binding protein disassembled actin filaments and is necessary for reorganize cell cytoskeleton and the formation of filopodia, controls cell migration. (19). Cofilin-1 is located at 11q13. High expression of Cofilin has linked with the invasiveness of different cancer cells (20), such as breast cancer, human prostate cancer (19), and malignant astrocytoma (20).

AXL is one of a Receptor tyrosine kinase family member and GAS6 has a high affinity for AXL (12). The AXL gene located on chromosome 19q13.2 (21). GAS6 is located at 13q34. AXL signaling promotes cellular invasion, migration, proliferation, and survival (12). AXL expression was associated with EMT (21). In metastatic ovarian tumor cells, AXL expression is dramatically increased and accumulating of these cells to form colony is noticeably depend on the GAS6/AXL signaling pathway (12).

GAS6, AXL, and Cofilin-1 are overexpressed, but because of tissue-specific functions of claudins family, Claudin-1 may have a different role in various cancers; depending on the origin of cancer cells and the adjacent tumor microenvironment, it may increase or decrease in various cancers. However, the relationship between GAS6, AXL, Cofilin-1, Claudin-1 expression levels, and various clinicopathological parameters has not been reported in detail. In the present study, the researchers investigated the relationship between different clinicopathologic features, as well as the relationship within the expression rate of four target genes by statistical tests.

## Materials and Methods

The biological materials were provided by the Iran National Tumor Bank, founded by the Cancer Institute of Tehran University of Medical Sciences, for cancer research. Fresh frozen tumor and normal adjacent tissue of seventy-eight patients with epithelial ovarian cancer who underwent surgery at the Cancer Institute of Iran were examined in this study. All samples were transferred from Tumor bank and stored at −80 ºC for further investigations. Sample data including patient history, histology, clinical, and paraclinical data were recorded.

All participants had assigned written informed consent. This descriptive study was approved by Ethics Committee of Tabriz University of Medical Science with TBZMED.REC.1394.1143 Code of ethics.

Total RNA was extracted from homogenized 50μg of tumors and normal tissues, using tripure reagent (Roche, Mannheim, Germany) according to the manufacturer’s instructions. Quantity and quality of prepared RNA were examined by Nanodrop spectrophotometer ND-1000 UV-Vis (Nano-Drop Technology) and running the RNA in agarose gel. The cDNA was synthesized from 1ug of total RNA, using TaKaRa cDNA synthesis kit (TaKaRa, Japan) with a random hexamer as a reaction primer in a final volume of 20 ul following manufacturer’s protocol. The cDNA quality was confirmed by the amplification of housekeeping gene glyceraldehyde3-phosphate dehydrogenase (GAPDH).

Quantitative determination of mRNA levels of target genes was performed in duplicate using SYBR Green I Premix (Takara, Japan) and 1ul of cDNA according to the manufacturer’s instruction in 40 cycles of amplification. The reaction conditions were 10 min of denaturation and enzyme activation at 95 °C followed by denaturation at 95 °C for 10 sec, annealing at 55 °C for 30 sec, and extension and florescence acquiring at 72 °C for 15 sec. The Rotor-Gene TM 6000 machine (Corbett Life ScienceTM, Germany) was used for this purpose. GAPDH was used as an internal control to normalize the target genes. CT was determined and then dCT was calculated by normalizing with the housekeeping gene. The used Oligonucleotide primers are listed in Table 1 Expression of four target genes (i.e., AXL, GAS6, Claudin-1, and Cofilin-1) was investigated in all tissues by using quantitative real-time reverse transcription PCR (qRT-PCR).

**Table 1:** Primer’s sequences

***Gene***	***Forward Primer***	***Reverse Primer***	***Size***
AXL	5′-GCCAGTGGCATGGAGTATCT-3′	5′-TACGTCCCTGGCGGTAGTAG-3′	154
GAS6	5′-AGCTGAGTTTGACTTCCGGA-3′	5′-CTTGATGACCAGATTCCGCG-3′	223
Cofilin1	5′-ATGCCCTCTATGATGCAACC-3′	5′-GCTTGATCCCTGTCAGCTTC-3	153
Claudin1	5′-TCGATACAATGGCACAGTGG-3′	5′-CAATCCCGCTATTGTGGTTT-3′	182
GAPDH	5-GAAGGTGAAGGTCGGAGTCA-3	5-AATGAAGGGGTCATTGATGG-3	109

GAPDH was used to normalize the gene expression. Then, the correlation with the expression level of target genes and Clinicopathologic Features (i.e., stage, grade, age, size) of ovarian cancer patients was investigated.

### Statistical procedures

SPSS software ver. 16 (Chicago, IL, USA) was used for statistical evaluation. The qRT-PCR data analyzed normality via the Kolmogorov–Smirnov method which failed (*P*<0.05); therefore, nonparametric methods (Mann–Whitney test, Kruskal–Wallis, and Spearman’s rank correlation) were used for statistical analyses. *P*-value <0.05 were considered as significant.

## Results

**Fig. 1:**
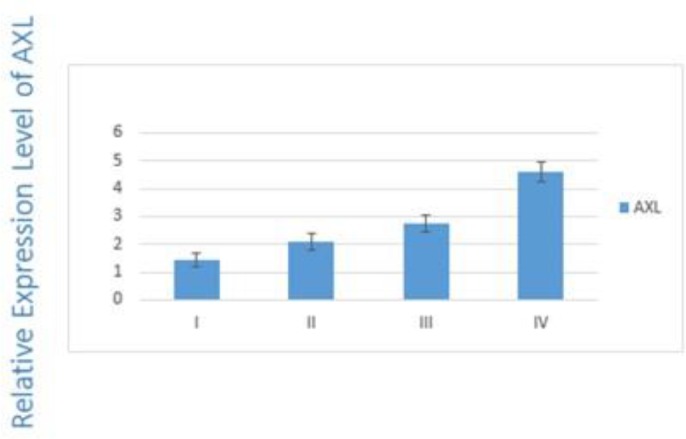
Expression of AXL in different stages

Table 2 shows the number of patients studied with regard to different considered clinicopathologic feature. According to the International Federation of Gynecology and Obstetrics (FIGO) classification, ovarian cancer has divided into four stages (i.e., I/II/III/IV). Results showed a significant correlation between the overexpression of AXL (*P*=0.03) and TNM staging (Fig. 1).

**Table 2:** Number of patient in different considered clinicopathologic feature

***Clinicopathologic futures***		***Number of patients***
Stage		
	I	23
	II	21
	III	21
	Iv	13
Grade		
	I	20
	II	20
	III	21
	IV	17
Size		
	≤7	10
	>7	68
Age(yr)		
	≤50	44
	>50	34

**Fig. 2:**
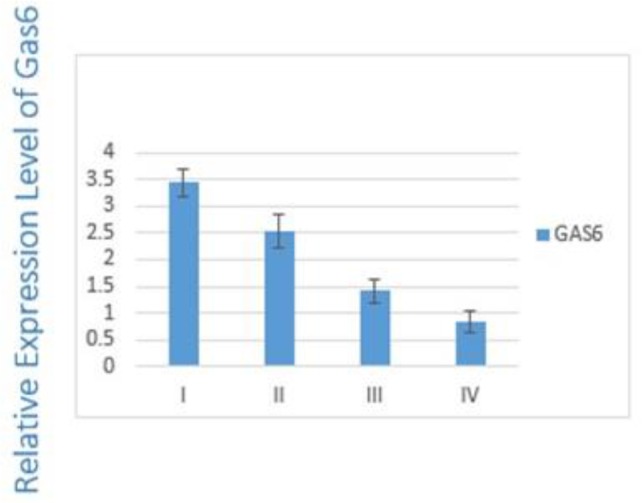
Expression of GAS6 in different stages

The differences between the stages I/III (*P*=0.01) and I/IV (*P*=0.03) were statistically significant. Figure 2 indicates the correlation between the expression level of GAS6 and TNM staging of disease. The expression level of GAS6 decreased in more advanced stages (*P*=0.01).

**Fig. 3:**
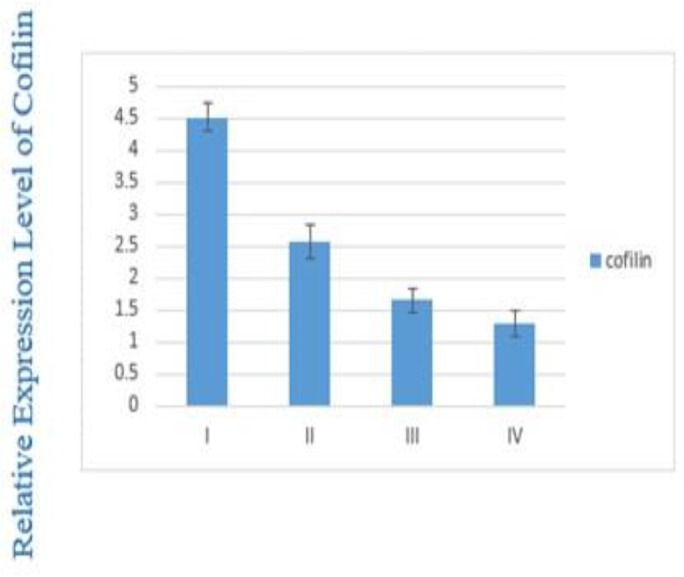
Expression of Cofilin1 in different stages

This decrease is significant between the stage I and IV (*P*=0.005) and stage II and IV (*P*=0.01). There is a negative correlation between Cofilin-1 expression level and TNM staging of disease (*P*=0.002) (Fig. 3).

**Fig. 4:**
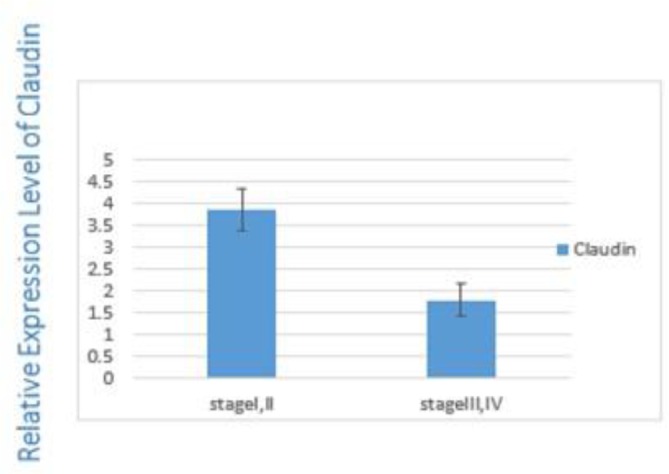
Claudin1 expression in low and high stage

This correlation is significant between the stage I/III (0.002), I/IV (0.001) and II/IV (0.03). There was a statistically significant difference between the expression level of Claudin-1 and TNM staging (*P*=0.01) (Fig. 4). Claudin-1 expression level was higher in low stages (stage I and II) compared with high stages (stage III and IV) (*P*=0.01) (Fig. 4).

There was no relationship between gene expression levels of AXL (*P*=0.6), GAS6 (*P*=0.5), Cofilin-1 (*P*=0.2) and Claudin-1 (*P*=0.3) with grades of tumor.

Size of tumor was another considered clinicopathologic feature classified into two groups of >7cm and ≤7cm. Expression levels of AXL and GAS6 were different in two major tumor size groups, although not statistically significant (*P*=0.7). Moreover, despite the overexpression of Cofilin-1 and Claudin-1 genes, the researchers did not find any significant relationship between tumor size and the expression of Cofilin-1 (*P*=0.2) or Claudin-1 (*P*=0.6).

In line with this finding, the age-specific rates of EOC, FSH, and LH/hCG enhance cell proliferation in primary human OSE and ovarian carcinoma cell lines (22). In this study, patients’ age range was within 15–83. Since the median onset age of ovarian cancer in Iran is estimated to be around 49–50 yr (3), patients were categorized into two age groups of ≤50 and >50. There is no significant relationship between AXL (*P*=0.8), GAS6 (*P*=0.1), Claudin-1 (*P*=0.5), and Cofilin-1 (*P*=0.9) gene expression levels and age in this study.

The correlation between target genes expression levels was evaluated in all patients. Cofilin1 and AXL have positive correlations with claudin1 and GAS6. And, GAS6 has positive correlations with AXL, Cofilin-1, and Claudin-1. Claudin-1 showed positive correlations with AXL, GAS6, and Cofilin-1 (Table 3).

**Table 3:** Correlations between target genes

			***Cofilin***	***Axl***	***Gas6***	***Claudin***
Spearman’s rho	Cofilin	Correlation Coefficient	1.000	−.091	.417^**^	.245^*^
		Sig. (2-tailed)	.	.428	.000	.031
		N	78	78	78	78
	Axl	Correlation Coefficient	−.091	1.000	.261^*^	.310^**^
		Sig. (2-tailed)	.428	.	.021	.006
		N	78	78	78	78
	Gas6	Correlation Coefficient	.417^**^	.261^*^	1.000	.385^**^
		Sig. (2-tailed)	.000	.021	.	.001
		N	78	78	78	78
	Claudin	Correlation Coefficient	.245^*^	.310^**^	.385^**^	1.000
		Sig. (2-tailed)	.031	.006	.001	.
		N	78	78	78	78

## Discussion

In this study, the expression levels of GAS6, AXL, Cofilin-1, Claudin-1, as genes involved in EMT, and their relationship with clinicopathological features include; age at diagnosis, TNM staging, size and grade of tumor were investigated in EOC patients.

A number of risk factors such as menstrual and reproductive factors, obesity, family history of ovarian cancer, and the recent decrease in population growth rate and parity, increase in the prevalence of overweight and obesity among Iranian women, associates with an increased risk of ovarian cancer (3, 23). Survival rates of patient are varied according to the stage of disease at diagnosis (24).

Women diagnosed at an early stage have a much higher survival rate than those diagnosed at a later stage; unfortunately, only 15% of patients with ovarian cancer are diagnosed in early stages and metastasis in tumor is the main problem in EOC (24). Therefore, fined tumor markers in order to improve diagnosis methods is a great challenge in the treatment of EOC (25). A crucial phenomenon correlates with the metastasis is EMT (9). Induction of transcription factors by signaling pathways such as TGFβ and (BMP), Notch, Wnt –β-catenin, Hedgehog, and receptor tyrosine kinases, changes the gene expression to promote loss of cell-junctions, cause the change in cytoskeleton and alteration from epithelial to the mesenchymal form (26). As the EMT associated genes are numerous, the researchers of the present study selected several up-regulated genes to demonstrate their potential applications as diagnostic markers (5).

In this study, results indicate that high expression of Cofilin-1 in patient sample tissues correlates with decreasing stage of ovarian cancer. In detailed statistical analysis, the differences between stages I/III, I/IV, and II/IV were significant. In another word, the expression level of cofilin-1 in the ovarian tumor was elevated in low stages (I, II) compared to that in high stages (III, IV).

The existing pieces of evidence suggest the crucial function of the cytoskeleton in EMT (9). Actin and associated proteins are changed and are necessary power to cell migration. The aggressiveness in neoplastic cancer cells correlates with the change in the regulation in cell migration (18). The Cofilin action has a crucial role in actin polymerization and constitution of cell membrane prominence for cell migration. The up-regulation of Cofilin is correlated with the aggressive phenotype of several tumor cells (20). Importantly, cofilin-1 play a role in multidrug resistance in pancreatic cancer, and platinum resistance in human lung adenocarcinoma cell lines, ovarian cancer, and tumor biopsies (27). Cofilin-1 expression level was significantly increased in breast cancer samples at TNM stages T0, T1 and T2 (based on tumor size (T stage)). There was only a slight increase that was not statistically significant at TNM stage T3 (28). Up-regulation of Cofilin-1 expression causes the progression of ovarian cancer (18). Aberrant cofilin1 expression was involved in cell invasion.

The finding of this study showed that Claudin-1 expression in stage II was higher than its expression in stages I, III, and IV in ovarian tumor sample, and a statistically significant correlation was between the Claudin-1 expression and tumor staging. Moreover, the differences in the expression level of Claudin-1 with in stage I/IV, II/III and II/IV were statistically significant.

Tight junctions have a significant role in keeping the cell polarity and also affect cellular transport (15). The claudins are necessary to the build-up tight junctions (TJs) in the epithelial and endothelial cell (13). Despite the role of claudins in the formation of mechanical cell adhesion at the site where epithelial and endothelial cells junc together, also, can recruit proteins involved in cell signaling. Thus Claudins are involved in the regulation of cell proliferation, differentiation, and consequent neoplastic transformation (29). Claudin-1 had a role in malignant progression of EMT (19). Moreover, had the potency to induction of EMT by interaction with signaling pathways and determined transcription factors (17). Tumorigenesis may result from the Mislocalization of claudin proteins (30). Claudins have a role in cancer progress via the interaction with different extracellular matrix molecules. In cloning research, claudin-1 increase matrix metalloproteinase-2 (MMP-2) activities by contact with membrane-type matrix metalloproteinase-1 (MT1-MMPs). This could increase the invasion ability of cancer cells by the decay of circumambient extracellular matrix ingredients, such as basement membrane, the claudin-1 overexpression in OSCC (oral squamous cell carcinoma) is correlated with, advanced stage and grade of the tumor (31). Claudin-1 up-regulated in metastasis tissues of colon cancer, by Mislocalization to the cytoplasm and cell nucleus (30). Claudin-1 expression decreased in breast cancers, while its high expression was proved in thyroid, urothelial, gastric and cervical tumors (31). The simplest define suggest claudin1 expression cause aggressiveness in a tissue-dependent behavior.

In this study, the AXL and GAS6 gene expression differences between tumor stages were statistically significant. GAS6 mRNA in ovarian tumor tissues was elevated more in stage I, II or low stages than stages III, IV. However, in the same tumor sample with overexpression of GAS6 (as a ligand), the expression of AXL (as a receptor) in stage I, II was lower than its expression in stages III, IV (high stages).

In the normal state, expression of Axl is negatively affected by GAS6. Although, under hypoxic condition, like tumors, hypoxia inhibits down-regulation of AXL by GAS6, consequently, tumor progression toward EMT may occur in cancers (7). AXL signaling activates with GAS6 in autocrine or paracrine form. Stress state in the tumor environment has a crucial role in the induction of GAS6/AXL signaling (32). In cells with prostate cancer, Axl expression in a hypoxic state not affected by the GAS6 so, it may be available for induction of an EMT-like condition that drives progression of metastatic condition (7). GAS6 is overexpressed in ovarian tumors. Moreover, GAS6 is overexpressed in glioblastoma and gastric cancers (25). Metastatic ovarian tumor cells are critically associated with the GAS6/AXL signaling pathway for metastatic colonize (12). Axl is overexpressed in pancreatic adenocarcinoma in stage II samples (7). The findings of the present research confirm the results of the recent studies about overexpression of GAS6 and AXL in tumor tissues.

## Conclusion

The present study indicates the up-regulation of AXL and down-regulation of GAS6, Coflin-1, and Claudin-1 in the more advanced stages of EOC. Given the importance of these genes in EMT process, alteration in these gene’s expression levels during the progression of disease may contribute to invasive behavior of cancer cells and distant metastasis of the tumor. Additionally, knowing the alteration pattern of these genes expression can help to better understanding and prediction of the prognosis of EOC.

## Ethical considerations

Ethical issues (Including plagiarism, informed consent, misconduct, data fabrication and/or falsification, double publication and/or submission, redundancy, etc.) have been completely observed by the authors.
